# Supplementary material for: The Anti-Metastatic Properties of Glutathione-Stabilized Gold Nanoparticles—A Preliminary Study on Canine Osteosarcoma Cell Lines
Source: Int J Mol Sci. 2025 Jun 25;26(13):6102. doi: 10.3390/ijms26136102 (PMC12249675; doi:10.3390/ijms26136102)
Supplement: Supplementary file 1 [file ijms-26-06102-s001.zip › ijms-3682584-supplementary.pdf]

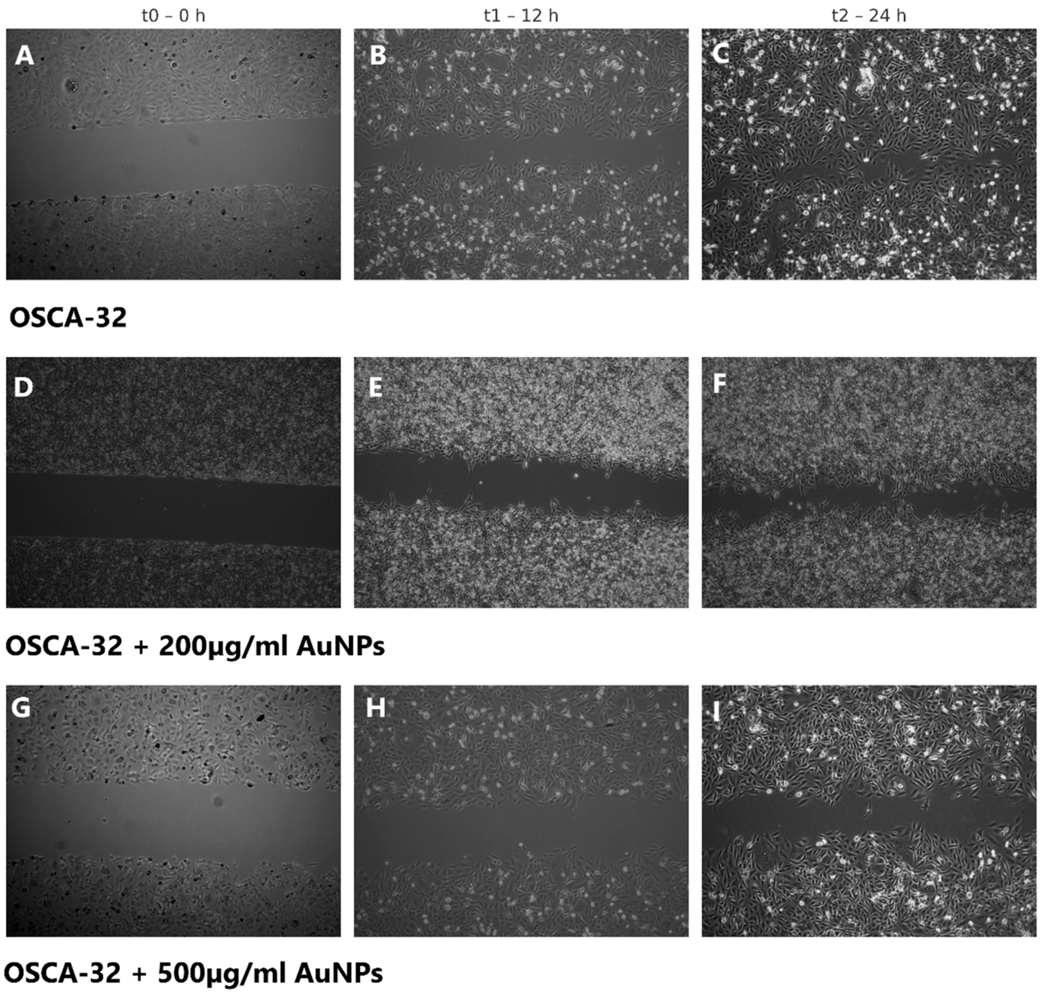

Figure S1. Wound-healing assay microscopic images showing the inhibition of canine OSCA-32 osteosarcoma cell migration by Au-GSH NPs. Untreated cells at t0 (0 h) (A); t1 (12 h) (B); t2 (24 h) (C); cells incubated with 200 µg/ml AuNPs at t0 (0 h) (D); t1 (12 h) (E); t2 (24 h) (F); cells incubated with 500 µg/ml AuNPs at t0 (0 h) (G); t1 (12 h) (H); t2 (24 h) (I).

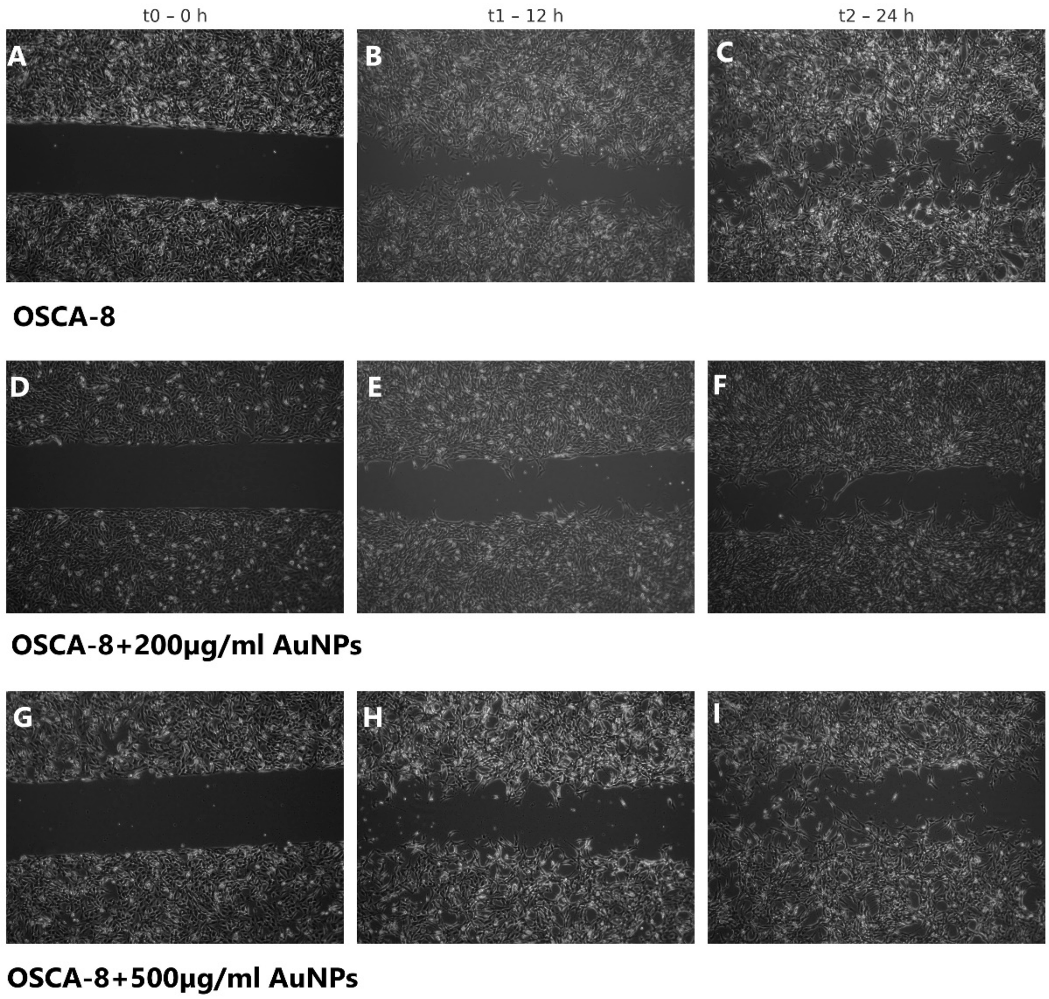

Figure S2. Wound-healing assay microscopic images showing the inhibition of canine OSCA-8 osteosarcoma cell migration by Au-GSH NPs. Untreated cells at t0 (0 h) (A); t1 (12 h) (B); t2 (24 h) (C); cells incubated with 200 µg/ml AuNPs at t0 (0 h) (D); t1 (12 h) (E); t2 (24 h) (F); cells incubated with 500 µg/ml AuNPs at t0 (0 h) (G); t1 (12 h) (H); t2 (24 h) (I).

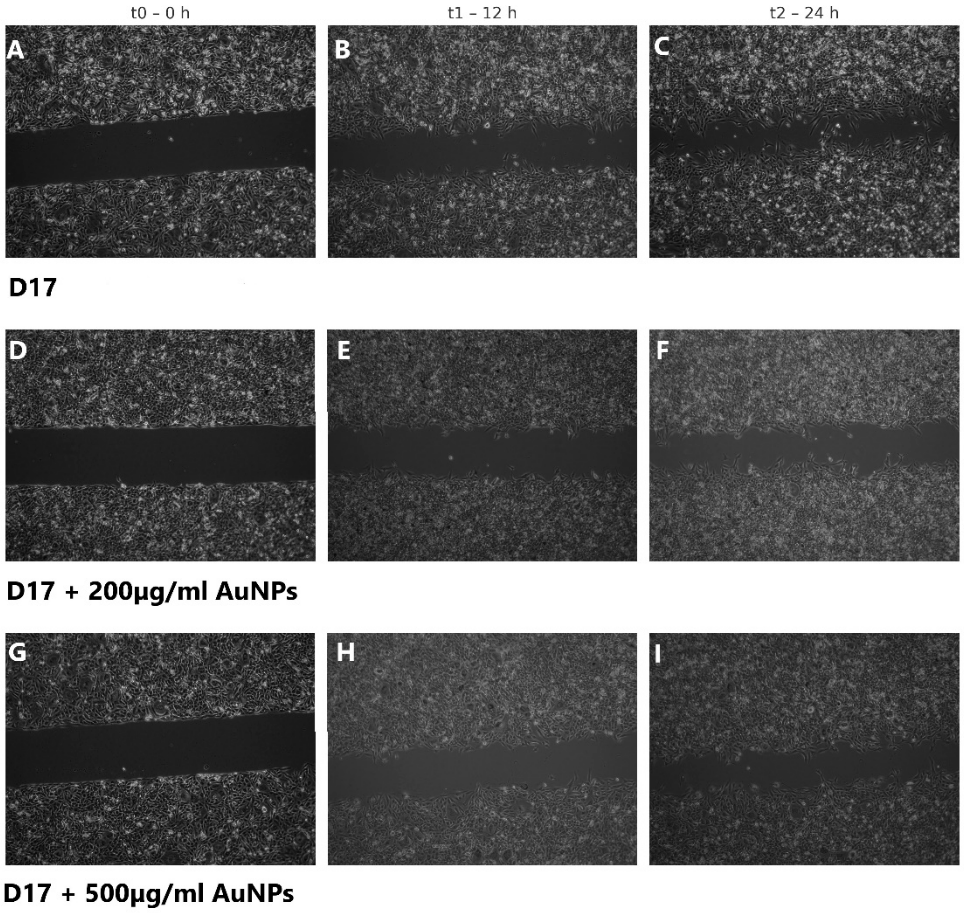

Figure S3. Wound-healing assay microscopic images showing the inhibition of canine D17 osteosarcoma cell migration by Au-GSH NPs. Untreated cells at t0 (0 h) (A); t1 (12 h) (B); t2 (24 h) (C); cells incubated with 200 µg/ml AuNPs at t0 (0 h) (D); t1 (12 h) (E); t2 (24 h) (F); cells incubated with 500 µg/ml AuNPs at t0 (0 h) (G); t1 (12 h) (H); t2 (24 h) (I).

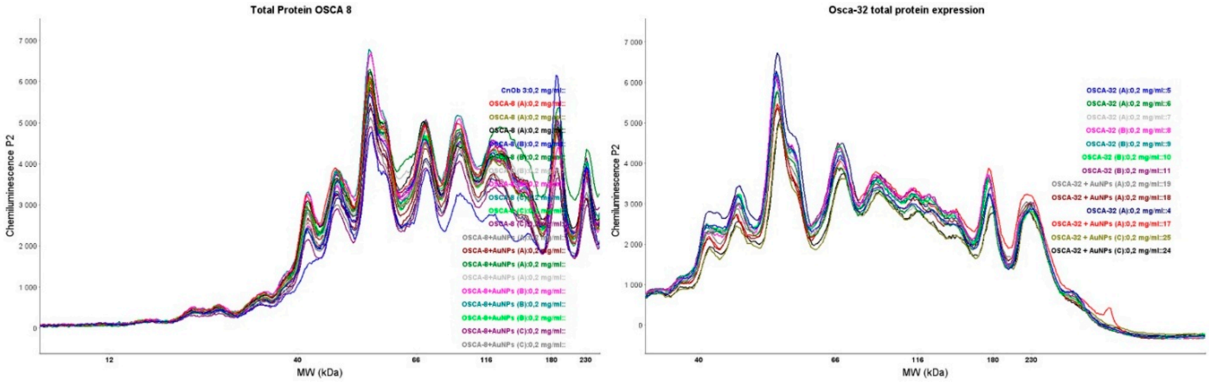

Figure S4. Total protein expression in OSCA-8 and OSCA-32 cell lines after AuNP treatment. Representative chemiluminescence profiles of total protein extracts from OSCA-8 (left) and OSCA-32 (right) cell lines treated with glutathione-stabilized AuNPs (0.2 mg/ml) in different formulations (A, B, C). Controls include untreated cells and CnOb-treated cells. Protein expression was analyzed using the Jess™ Simple Western system.
